# Contextualized understandings of dairy farmers’ perspectives on antimicrobial use and regulation in Alberta, Canada

**DOI:** 10.3168/jds.2021-21521

**Published:** 2022-11-21

**Authors:** Jennifer A. Ida, Warren M. Wilson, Daryl V. Nydam, S. Craig Gerlach, John P. Kastelic, Elizabeth R. Russell, Kayley D. McCubbin, Cindy L. Adams, Herman W. Barkema

**Affiliations:** 1 Department of Production Animal Health, Faculty of Veterinary Medicine, University of Calgary, Calgary, AB T2N 4N1, Canada; 2 Department of Population Medicine and Diagnostic Sciences, Cornell University, Ithaca, NY 14853; 3 Department of Anthropology, University of Calgary, Calgary, AB T2N 4N1, Canada; 4 Faculty of Environmental Design, University of Calgary, Calgary, AB T2N 4N1, Canada; 5 Animal Welfare Program, Faculty of Land and Food Systems, University of British Columbia, 2357 Main Mall, Vancouver, BC V6T 1Z4, Canada; 6 Department of Veterinary Clinical and Diagnostic Sciences, Faculty of Veterinary Medicine, University of Calgary, Calgary, AB T2N 4N1, Canada

**Keywords:** antimicrobial use, behavioral context, ethnographic fieldwork, policy, dairy farmers

## Abstract

Antimicrobial resistance (AMR) has been largely attributed to antimicrobial use (AMU). To achieve judicious AMU, much research and many policies focus on knowledge translation and behavioral change mechanisms. To address knowledge gaps in contextual drivers of decisions made by dairy farmers concerning AMU, we conducted ethnographic fieldwork to investigate one community’s understanding of AMU, AMR, and associated regulations in the dairy industry in Alberta, Canada. This included participation in on-farm activities and observations of relevant interactions on dairy farms in central Alberta for 4 mo. Interviews were conducted with 25 dairy farmers. The interviews were analyzed using thematic analysis and yielded several key findings. Many dairy farmers in this sample: (1) value their autonomy and hope to maintain agency regarding AMU; (2) have shared cultural and immigrant identities which may inform their perspectives of future AMU regulation as it relates to their farming autonomy; (3) feel that certain AMU policies implemented in other contexts would be impractical in Alberta and would constrain their freedom to make what they perceive to be the best animal welfare decisions; (4) believe that their knowledge and experience are undervalued by consumers and policy makers; (5) are concerned that the public does not have a complex understanding of dairy farming and, consequently, worry that AMU policy will be based on misguided consumer concerns; and (6) are variably skeptical of a link between AMU in dairy cattle and AMR in humans due to their strict adherence to milk safety protocols that is driven by their genuine care for the integrity of the product. A better understanding of the sociocultural and political-economic infrastructure that supports such perceptions is warranted and should inform efforts to improve AMU stewardship and future policies regarding AMU.

## INTRODUCTION

Use of antimicrobials plays a major role in prevention and control of infectious diseases in food-producing animals including dairy cattle (Tang et al., 2017). There is, however, evidence that antimicrobial use (**AMU**) in livestock contributes to selection of novel strains of bacteria with antimicrobial resistance (**AMR**) that have the potential to cross over to human pathogens (Laxminarayan et al., 2016; Robinson et al., 2016; Wall et al., 2016). There are also multidrug-resistant genes in many shared human-animal-environmental pathogens (Robinson et al., 2016). Direct links between AMU in animals and AMR in humans remain unclear (Tang et al., 2017), with some regarding the association as uncertain or indirect (Wall et al., 2016; Tang et al., 2017). Other studies consider food animals a route of exposure (Woolhouse and Ward, 2013) and environmental sources (manure spread, hospital waste, water treatment plants, and so on) as important contributors (Woolhouse and Ward, 2013; Laxminarayan et al., 2016; Robinson et al., 2016; Wall et al., 2016).

Given potential associations between AMU on farms and emergence of AMR strains of bacteria in livestock and humans, research and policies have focused on knowledge translation and behavioral change mechanisms to reduce AMU across all health sectors (Raymond et al., 2006; Friedman et al., 2007; Charani et al., 2011), with only marginal success. For example, although farmers in Washington state concluded that educational interventions reduced AMU, objective analyses indicated no post-intervention reduction in AMU (Raymond et al., 2006). These interventions may be compromised by the assumption that people are rational actors (Jansen and Lam, 2012), despite an abundance of social and veterinary science findings to the contrary (Kahneman and Tversky, 1979; Thaler, 1980; Tversky and Kahneman, 1981; Jansen et al., 2010; Lam et al., 2011). Decisions arise from a complex and dynamic mix of reason and intuition, informed by context, biology, and anticipated outcomes (Thaler, 1980; ESRC Working Group, 2014; Sapolsky, 2017). Therefore, abundant evidence from experts regarding AMU may have limited effect on behavior if local perspectives are ignored (Craig et al., 2018). The efficacy of policy guided by local context is evident in the Netherlands, where the RESET Mindset Model, which incorporated local perspectives on AMU among farmers, in combination with AMU reductions required by legislation, achieved a sustained 47% decrease in AMU between 2009 and 2015 (Lam et al., 2017).

Despite substantial literature on farmers’ knowledge, attitudes, and practices concerning AMU (Hockenhull et al., 2017; Higham et al., 2018; Parkunan et al., 2019; Vasquez et al., 2019; Chan et al., 2020) and a rapidly growing body of literature on the importance of collective systems in AMU decision-making in other farming sectors (e.g., broiler chickens; Adam et al., 2020), there is still a paucity of literature that provides an in-depth understanding of the context in which AMU decisions are made or that looks beyond behavior of individual dairy farmers. Some exceptions include work conducted with dairy farmers in Sweden, Ontario (Canada) and the UK, which acknowledge the importance of context, recognizing that behavior is built upon a socially-constructed foundation (Cobo-Angel et al., 2021; Cobo-Angel et al., 2022; Fischer et al., 2019; Rees et al., 2021b). However, contextualized understandings of dairy farmers’ perspectives on AMU and prospective regulation in Alberta have yet to be considered. To begin to address this knowledge gap and inform public policy, the objective of this study was to elucidate perceptions of one community of Albertan dairy farmers concerning AMU and AMR within their broader context using anthropological methods.

Antimicrobial resistance is at the human-animal-environmental nexus, rendering it a quintessential One Health issue (McCubbin et al., 2021), best informed by synthesis of input from biomedical and social sciences (Zinsstag et al., 2009; Craddock and Hinchliffe, 2015; Brown and Nading, 2019). Many people, including farmers, veterinarians, and physicians, hold deeply ingrained beliefs that guide AMU practices (ESRC Working Group, 2014). As noted above, effective, sustainable AMU policy must be informed by an understanding of how antimicrobials are perceived and used within target communities (ESRC Working Group, 2014). As other studies have noted, effective stewardship cannot be separated from the contexts in which practices are occurring (Kirchhelle et al., 2020; Rees et al., 2021a). Anthropology has the potential to provide such context via ethnographic fieldwork (Vayda, 1983). Such fieldwork entails prolonged, immersive interactions with the people of interest that can, in turn, generate an understanding of variables affecting behavior (Schultz and Lavenda, 2009). In this study, anthropological immersion by the first author (JAI) into the lives of those interacting with, caring for, and medically treating dairy cows, provided insights on the context shaping on-farm AMU with the potential to guide relevant policy.

## MATERIALS AND METHODS

This study was reviewed and approved by the University of Calgary Conjoint Faculties Research Ethics Board (study ID number REB18–0795).

### Positionality Statement

Because ethnographic fieldwork draws together individuals with distinct cultural backgrounds and places them in a context together (Schultz and Lavenda, 2009), it is important to understand the identity and the culture of the researcher. The background of the researcher has shaped her worldview and consequently had some effect on her interactions with community members as well as her interpretation of the information gleaned through the fieldwork.

At the time of the study, the first author, JAI, was 30 years old. She was an MSc student in the Department of Production Animal Health in the Faculty of Veterinary Medicine at the University of Calgary. She previously completed a master’s in anthropology at University of Colorado Boulder. Before that, she completed a bachelor’s at Wagner College in New York, New York, where she majored in anthropology and minored in microbiology and Spanish. Her multidisciplinary background spans the natural and social sciences and so made her uniquely positioned to carry out this project. She was born and raised in Staten Island, New York, a borough of New York City, New York. Although having grown up in a politically conservative borough, she identifies as liberal. At the time of the study, she had some clinical veterinary experience, but no previous experience with dairy cattle, farming, or medicine in intensive farming systems. Because she is not from an agricultural background, she was able to begin her fieldwork as a nonexpert, acknowledging the importance of the farmers’ knowledge and experience. She culturally identifies as Italian American. She was raised Catholic, although at the time of the study did not regularly engage in religious activities. Her parents refrained from overuse of Western medicine during her early childhood, promoted alternative forms of care and were particularly cautious about antibiotic use. Last, the researcher does not consume most dairy products due to a food intolerance and so is not currently a regular part of the consumer population.

### Characteristics of Dairy Farming in Alberta

At the time of this study, the Alberta dairy industry consisted of 512 dairy farms with an average of 156 cows per herd. Eighty percent of the dairy farms were family-owned and operated, 20% of the farms were part of a Hutterite colony (Alberta Milk, Edmonton, AB, Canada). On approximately all Alberta farms, cows were housed all year inside a barn, and on most (93%) of the farms, cows were housed in a freestall barn (Canadian Dairy Information Centre, 2020). The predominant cattle breed used was Holstein (Alberta Milk, AB, Canada).

### Sampling and Study Site

#### Field Site.

Ethnographic data were collected by the first author (JAI) over a period of 4 mo in a small agricultural community in south-central Alberta, with a population of approximately 7,229 (Census Profile, 2016). While there, JAI resided with a dairy farming family during several homestays (cumulation of 11 d in Summer 2018) before commencement of interviews and, subsequently, with a veterinarian who practiced at the local, mixed-animal clinic for a period of 3.5 mo (January–April 2019).

#### Source Population.

All dairy farmers in central Alberta, including immediate adult family members of the primary owners, were eligible. Study participants came from dairy farms within 135 km of the focal community. The observational sample also included other individuals involved in on-farm work, including milkers, hoof trimmers, veterinarians, and others in the broader community.

#### Sampling Strategy.

Author JAI used snowball sampling to recruit participants. Initially, co-author HWB introduced JAI to a dairy farmer. This farmer subsequently introduced JAI to their veterinarian and other dairy farmers. JAI also recruited participants by accompanying veterinarians on farm visits.

#### Sample Size.

Author JAI interviewed 25 dairy farm owners. In 10 (40%) of the interviews, children, parents, or in-laws also participated. JAI also engaged in informal discussions with 12 additional dairy farmers during herd health visits. These 12 dairy farmers were in addition to the 25 interviewed. Of the 25 dairy farmers interviewed, 7 (28%) were included in the participant and direct observation aspects of the study, based on participants’ interest. This resulted in approximately 3 d on each of the 7 farms (plus additional days that included farm tours of all 25 farms, lunch visits, caretaking of children, herd health exams, and the homestays). In addition, JAI also conducted participant and direct observation with veterinarians, hoof trimmers, on-farm employees (milkers, DHI personnel, AI or genetics company technicians), and other members of the broader community during 3 community events, 2 hoof trimming visits, 6 religious services with 2 congregations, 23 herd health exams, and 1 antimicrobial workshop.

### Data Collection

Three categories of ethnographic research were used: participant observation, direct observation, and open-ended interviews.

#### Participant Observation.

Participant observation, a key data collection method used in ethnographic fieldwork, requires the researcher to participate in daily chores, activities, and social events (Hammersley and Atkinson, 2007; Schultz and Lavenda, 2009). Consequently, the researcher is able to “absorb their mode of reasoning” and gain understanding of family dynamics and values (Opala and Boillot, 1996). In this study, observations occurred on dairy farms and at community events. Research settings included barns, farm fields, homes, community centers, and churches. This involved participating in daily farm routines, including milking cows, bottle-feeding calves, cleaning pens, and delivering feed, whereas home duties included childcare, cooking, and gardening. Author JAI also attended events hosted by a local community group (i.e., a pancake breakfast, hockey game, and barn tour). As well, after being invited by members of 2 congregations, JAI attended Sunday church services.

#### Direct Observation.

Author JAI also directly observed participants to understand behavior in context (Schultz and Lavenda, 2009) and to compare what people say and what they do (Gobo, 2008). Unlike participant observation, during direct observation, the researcher does not participate, but simply observes. In this study, direct observation consisted of observation of on-farm activities, including crop-related activities, treatment of sick cows and calves, surgeries, calf euthanasia, necropsies, calvings, milking procedures for cows given antimicrobials, dry cow treatment (DCT), hoof trimming, and herd health examinations. Observation was ~3 d on each of the 7 farms. Each daily visit lasted 4 to 8 h, depending on the farmer’s schedule and support. Additionally, JAI toured all 25 farms and a Hutterite colony where dairy farming occurred. Observations were recorded as fieldnotes.

#### Open-Ended Interviews.

Open-ended, semi-structured interviews were conducted (Bernard, 2011). The interview guide was reviewed by co-authors for suitability and tested and validated by a farmer external to the study to ensure that questions were appropriate. Interviews lasted 1 to 4.5 h, (average, 1.6 h). Key topics included, but were not limited to concerns and perceptions regarding reductions in AMU and increased regulation of antimicrobials, perceptions of AMR, and perceived risks of adopting new protocols. Areas for increased antimicrobial stewardship and potential pathways for collaboration with veterinarians and hoof trimmers were discussed. Additionally, information regarding belief systems and lived experiences was gathered in interviews. Participants each received an anonymous participant ID and are referred to in the paper as **P#**.

#### Fieldnotes.

During the initial homestays, fieldnotes were recorded each day. During the latter data collection period, fieldnotes were recorded approximately every 4 d.

### Analyses

#### Analysis of Interviews.

Interviews were audio-recorded, then transcribed by a professional transcriptionist. Thematic analysis was used to analyze the 25 interviews (Bernard et al., 2017). The thematic analysis was conducted in 4 broad steps:

1. Familiarization with the data: Transcripts and fieldnotes were reviewed by JAI; audio recordings were listened to closely; transcripts were verified against audio recordings; and detailed notes were recorded for each interview and summarized.
2. Coding and organization: Codes were identified as phrases, or a few words, that described values and beliefs regarding AMU, AMR, and regulation (Bernard et al., 2017). JAI coded the first 9 transcripts and formed the basis of the original codebook. The original codebook was applied to the additional 16 transcripts by ERR, KDM, and JAI using NVivo (QSR International Pty Ltd. Version 12, Burlington, MA). ERR, KDM, and JAI discussed the codebook, emergent themes, and reviewed for intercoder reliability. Disagreements and suggestions for altering the codes were discussed by coders and the codebook was adapted accordingly. Following this process, the first 9 transcripts were reviewed using the final codebook.
3. Generation of themes: Ideas generated from the codes were grouped into themes and subthemes and further organized by JAI and HWB, following a latent, rather than semantic approach. That is, themes were not solely descriptive in nature, but also took into consideration underlying ideas potentially informing participants’ responses.
4. Reporting: Quotes were extracted to provide concrete examples of themes.

#### Analysis of Ethnographic Data.

Fieldnotes based on observations and informal discussions were transcribed from handwritten fieldnotes and organized to contextualize and inform interview data and to provide a holistic account of the social context of AMU by the dairy farmers of this region (Gertz, 1973). The ethnographic fieldwork served as a means by which to understand the most salient findings of the interviews and how the perceptions described in the interviews related to the values, lived experience, and broader context of the producers.

## RESULTS

### Descriptive Statistics

All farms in this sample had connections to dairy farming through family lineage (with at least 3 extending back <4 generations and 1 to the 17th century; Table 1), many of which had either direct European heritage (immigrated from the Netherlands or Switzerland) or were descendants of European immigrants (parents, grandparents, or great-grandparents from the Netherlands or Switzerland). All farmers in this study identified their farms as nonorganic, family farms. Additional data on participating farms and producers provided in Tables 1 and 2.

### Context

An overview of the context is described below to provide further understanding of the lived experience of the farmer in this particular dairy farming community.

### Supply Management System

A distinguishing feature of the Canadian dairy industry is supply management. Throughout interviews, farmers frequently mentioned supply management, sharing how they have fought to retain it, as it provided economic stability. One farmer noted that the stability guaranteed by supply management emboldened him to take on greater challenges and risks (e.g., making changes to housing).

### Access to Antimicrobials

Current AMU regulation is a complex mix of national and provincial laws as well as local by-laws (Canadian Veterinary Medical Association, 2021b). Current antimicrobial policy for dairy farmers in Alberta allows farmers to practice blanket DCT, maintain an on-farm pharmacy, and administer antimicrobials. Use of ceftiofur is permitted. As of December of 2018, new regulation stipulated that antimicrobials could no longer be purchased over-the-counter at feed stores and a valid veterinarian-client-patient relationship must be established before the sale of antimicrobials (Canadian Veterinary Medical Association, 2021a).

### Religion

Although not all individuals in the dairy farming community are religious, religion is clearly part of the social fabric of this context. Of those who participate in religious activities, many were Dutch Reformed and worshiped each Sunday. After services, there were opportunities to meet other members of the congregation, reflect on the service, and strengthen community ties. As described by one participant, religion and social relationships are often linked in rural communities. Two participants shared that once Dutch dairy farmers immigrated to rural Alberta, they built their own communities and as one participant explained, because they were far from home and far from their families, the church served a familial role.

For participants who expressed strong ties to the religious community, their beliefs also influenced their approach to farming and laid the groundwork for on-farm decisions and practices. One participant explained, “When it comes to me and my worldview and my understanding for why [I] farm and how farming fits into my picture of society, it is ultimately, for me, a theological worldview that I use to answer those questions. So, it will be that I am commanded, we are commanded, to treat everybody with dignity and respect and take care of people. So, that means, in farming, I have to treat my employees with dignity and respect…and animals, they’re God’s creation. They are not mine to exploit” (P16). The same participant later connected his regard for animals to AMU. He stated, “For us, the Bible says, ‘The righteous man regards the life of his animal, takes care of his animal.’ …It’s a moral question, ultimately. Antibiotic use, to me, is ultimately a moral question.” Another farmer used his biblical understanding to state his identity, and the collective identity of farmers, as stewards of the land. “I think the Bible is very specific [that] we are stewards of the land. And, our job is to take care of [the land]…” (P15). Yet another participant highlighted the farmer’s role as a steward and drew a connection to the stewardly use of resources, specifically AMU. He stated, “[God] has given us abilities. He has given us resources. Our perspective is that we are supposed to use those resources and that we are supposed to be good stewards of them…If you are using more than you really need to in order to treat your sick animals, or if you are being excessive with it, then I would say that’s morally wrong” (P18).

### Technology

Participants embraced technological innovation and strived for constant on-farm improvement. The focus on technological innovation was made clear through the ethnographic observation both on-farm and in regional conferences such as the Western Canadian Dairy Seminar (WCDS; Red Deer, AB, Canada). The first author’s initial introduction to the community and the field site was at WCDS, a farmer-focused research symposium. However, rather than walking into an auditorium filled with only research posters, the space was filled with individual vendors that offered novel technologies to improve on-farm efficiency and management. Farmers were observed visiting the booths and learning of new products on the market, while also conversing with other farmers about the products and new updates on their individual farms.

During the fieldwork, JAI was invited to an on-farm social gathering often held after a recent technological installation, or barn renovation. In this particular case, the host farmer had recently switched from a milking parlor to an automated milking system. He gave an informal tour, describing unique features and comparing it to other models. The high importance given to technological innovation was also evident during interviews, in which farmers discussed estrus detection, rumination, and activity monitors, as well as the wide range of data collected from these systems and how that improved decision-making, particularly as it related to AMU. During an interview, one participant shared, “I think if you want to use less antibiotics you have to invest in technology to know more about your cows” (P7).

### Family and Heritage

#### Multigenerational Farming.

As noted above, for most of the farmers in this study, farming is part of their cultural heritage—in either Canada, the Netherlands, or Switzerland (see Table 1). Most farmers are part of a farming tradition that extends back several generations, and most current operations involve contributions of multiple family members. Many of the farmers in this sample still maintained close ties to Holland via family and friends, many of whom were still involved in dairy farming. One farmer said, “I’ve been watching this field very carefully from Holland. That’s my country of origin. I still have family that farms there” (P16).

#### Europe to Canada.

Dairy farmers who immigrated from Switzerland or the Netherlands did so with the intention to farm. One farmer who immigrated from the Netherlands stated, “I left the Netherlands because I was a small farmer in the space of trying to [develop] myself…You have competition in Holland from big farmers, and the regulations there, they got tougher and tougher. So, at the end, we decided we go to Canada. And not because we want to escape only the regulations, because we can handle regulations…But it’s more [that] we have more. Now the boys have a chance to farm” (P5). Others mentioned that farmers in the Netherlands felt increased pressure due to increasing regulations, which no longer made dairy farming enjoyable. One farmer who relocated from Switzerland said he too moved to Canada to farm, as “You can’t farm in Switzerland if it’s not in the family pretty much…You just can’t afford it” (P4). Another farmer from Switzerland shared, “In Switzerland, there’s no opportunity to farm anymore because there are too many government regulations. You farm for subsidies. You’re not profitable without subsidies. It is terrible and the rules are made by urban dwellers. And, they’re not feasible or economical half the time. And, the animal welfare requirements are unrealistic. And, it’s just, it became too much of a hassle” (P10).

### Built Environment

A key aspect of the built environment is farm distribution; dairy farms in Alberta were described by participants as family farms that are geographically dispersed. According to farmers in this sample, the built environment in Alberta makes AMU policies, such as those implemented in some European countries which require the veterinarian to provide all on-farm medical treatments, impractical in this context. One farmer stated that the Netherlands and Denmark are, “smaller countries. I think it is easier to get around. I think Canada or the US…, we’re more spread out” (P1). Given the nature of the built environment, farmers were concerned about potential delays in time-sensitive medical treatment should future regulation require a veterinarian for administration of antimicrobials.

### Themes and Subthemes

Five themes emerged from the thematic analysis. These were: (1) Values; (2) Lack of trust and misguided information; (3) Dairy farmers’ perspectives on AMR, (4) Dairy farmers’ perspectives on antimicrobial regulation; and (5) Dairy farmers’ perspectives on current AMU and stewardship.

### Theme 1: Values

#### Subtheme 1a: People Who Care.

Several key values were expressed by dairy farmers throughout the interview process and observed during the fieldwork. Dairy farmers in this sample can be described as people who care. They care about animal health and welfare, the environment, people and their community, and they take great pride in the safety and integrity of their product. One farmer shared, “Built into everybody is a genuine care for people. We are human beings that care” (P16).

#### Subtheme 1b: Dairy Farmers as Autonomous Actors.

When asked about the impetus behind the desire to farm, participants offered a variety of responses including ones that pertained to the lifestyle that farming provides, a lifestyle that allows for time with family, stability, and for the individual to be creative, inventive, and challenge oneself. However, when asked what they valued most about being a dairy farmer, most responded with a version of, “I am my own boss” or simply, “my freedom.” For example, one farmer exclaimed, “You have all that freedom, right? Being your own boss” (P24). And yet another farmer shared, “It is a lot of us farmers because we like doing things our own way and we don’t like having anybody to really answer to. That’s kind of farm mentality, ‘It’s my farm. [I will] figure it out. I am going to do it my way’” (P15).

This value was explicitly reflected in concerns related to AMU regulation: “I hope [antimicrobials] will always stay available to us…You try to reduce as much as you can, as long as it is still available so you can act when you think you need it…” (P7). This freedom provides them with a sense of agency (i.e., the ability to act as they see fit). This desire to remain autonomous applies to authority figures, and includes their veterinarians, as described by their concerns about the potential reliance on their veterinarian that specific AMU regulations would create (described in Subtheme 4a). However, strict independence from the veterinarian is not desired. The role of the veterinarian is significant in this context and discussed below (Subtheme 5c).

### Theme 2: Lack of Trust and Misguided Information

#### Subtheme 2a: Perceived Mutual Distrust Between Farmers and the Public.

Many farmers expressed frustration regarding both the general public and consumers’ perceptions of dairy farming. The general public, as referred to by the study sample, often, but not always refers to non-agricultural, urban dwellers. Consumers comprise a subset of the general public and therefore overarching societal perceptions are likely reflected in consumer ideology. Some feel unfairly blamed for environmental and health issues that affect society on a global scale. One stated that “Every major city in North America dumps more raw sewage into our water ways than all the farms across the entire continent…But do we address that problem? No! It’s the farmer’s fault. And farmers all across North America are … tired of being blamed for urban areas’ problems” (P3).

According to one farmer, “I think some of the farmers have lost a little trust in the consumers, and the consumers maybe lost a little bit of trust in the farmers…” (P6). He also provided a somewhat contradictory statement, suggesting he may be conflicted or hold multiple viewpoints when he added that “Generally, I think people do trust us to [do] a good job and [provide] a safe, wholesome product.” Concerned about the gap between farmers and consumers, he stated, “We have to close that gap between people consuming milk products and what we’re actually doing on the farm” (P6). Unfortunately, he and other farmers are not optimistic about achieving that outcome. Farmers noted the difficulties in trying to change consumer perception, despite repeated efforts to cultivate a positive image for the industry and to assure consumers of milk quality and safety. Additionally, farmers had increased frustration, particularly where they consider the consumer unable to see the “big picture” or misguided by antibiotic-free claims of other products, social media, and influential groups, which portray conventional farmers in a negative light.

The same farmer also stated that constant public criticism created an environment in which the producer is not open to change, but rather becomes more committed to his position. He shared, “…if we get criticized long enough, you kind of get more radical with your stance, defending what you are doing instead of looking for a new approach to maybe change” (P6). He felt that this was unfortunate and occurring in many farming sectors. “We are so in the public eye now… A lot of times, you are kind of that monster who takes away the calves from their moms, has antibiotics, hormones, and pus in their milk…” (P6). He also described the difficulty in balancing the interests of the public against the welfare of the animal, drawing attention to consumer perceptions of the word “antibiotic.” He shared: “Even when you try to tell [the consumer you are treating to address] a clinical problem and [doing it] for the betterment of the animal, it’s still that word, [antibiotic], that’s out there and kind of harmful…” (P6). He weighed the 2 options. One option, he explained, would be to continue with current treatment practices and ensure that his animals are safe and the other is to “radically change” and trust that the public acknowledges this change. After weighing his options, he concluded, “But, I definitely think it’s important to really start a discussion about [antibiotic stewardship]… how it should happen, what we can really do… to make sure the animal is still safe” (P6).

Several farmers noted the growing detachment of the public from farms, noting most consumers have limited understanding of farm activities. Therefore, if policy makers implement new regulations that require reductions in AMU, farmers are dubious that consumers will be aware of the changes or that it will improve consumer perception of farmers.

We detected a view that consumer perceptions are essential, as without the consumer, there is no market. One farmer said, “As an industry…we’re always trying to get our message out. We do our due diligence. We want to make sure that, you, the consumer, [is] happy. And we’re always looking for consumer feedback” (P1). Although some regarded it as difficult or impossible to gain consumer trust, to others, it was critical.

#### Subtheme 2b: Perceived Lack of Value for Farmer Knowledge, Experience, and Education, as Further Exacerbated by Implementation of “Extreme” Policies.

Farmers expressed frustration and disappointment with public perceptions, specifically in terms of their knowledge, education, and experience. Although generally supportive of reducing AMU to protect human health, one farmer, concerned about policy that would require a veterinarian to treat his cows, stated, “I think it totally takes away the experience and knowledge that the farmers already have… You are…essentially being told that you do not know what you are doing, and you just need to conform… If that were to happen, it would be very disappointing. I am sure there would be a better way to do it” (P2). Yet another farmer shared a similar perspective, but specifically mentioned frustration with consumer perceptions. He stated, “I think a lot of the consumers think farmers don’t know what they’re doing, they’re not educated, and that a lot of the non-farming people know better what’s supposed to be done than the farmers themselves. I think that’s a problem…I hope people understand that a lot of farmers are educated, and they do get training, right?” (P7). Yet another producer stated the need for public education regarding farmers as people, namely who they are and how they make decisions, while another shared his confidence in his ability to observe a cow’s clinical signs and treat accordingly.

### Theme 3: Dairy Farmers’ Perspectives on AMR

#### Subtheme 3a: Views of the Connection Between AMU in Agriculture and AMR in Humans.

Due to diligent following of mandatory milk safety protocols (i.e., withdrawal times, bulk tank testing for antimicrobial residue), some farmers were unclear on how dairy farming could contribute to AMR in humans with such protective measures already in place. Regarding changing on-farm AMU practices that may be linked to AMR in people, one farmer responded, “If I have proof that people are consuming milk and they are getting resistance to antibiotics because of it, maybe I’ll change my tune. But I do not believe that’s happening” (P3).

Others were less skeptical of the connection, did not consider consumer concerns to be unreasonable, or were very aware of AMR, at least as it pertained to their herd. To prevent AMR, one farmer treated with a maximum of 2 antimicrobials prior to culling and was very clear on decision-making regarding treatments, culling, communication, and expectations with employees regarding treatments and milking. This farmer stated, “If it’s *Staph*, it’s never going to be fixed, so you give a cow a chance, but not continuously trying again and again. I think that’s important to me. And that’s how you build resistance too” (P7). Others noted a generational discrepancy in terms of awareness regarding the topic, with younger farmers being more cognizant of growing global health issues, compared with their parents. One farmer said, “I think there’s a bit of a generational difference, too, though. Me and [my husband’s] generation, we’re more concerned about those issues than my parents’ and [his] parents’ generation” (P5).

#### Subtheme 3b: Lack of Clear Information Surrounding Pathways of AMR and Connection to Milk Safety and Procedures.

Farmers were frustrated by a lack of clear information regarding the pathway by which AMU on dairy farms may pose a public health concern. One farmer shared, “…they act like we’re shipping milk and meat with antibiotics in [it]. I’m kind of like, ‘No, there’s not. You’re not allowed to do that.’ And, in the milk, it doesn’t work because you can’t make cheese, you can’t make yogurt, you can’t make anything. So, there are no antibiotics in it” (P9). Participants take great pride in meeting Canadian standards. Many responded with arguments that milk was safe and free from antibiotic residue; they can attest to this because when they administer an antimicrobial to a lactating cow, they must adhere to strict withdrawal times or risk hefty penalties. One farmer stated, “…we don’t have issues with residues because we have withdrawal periods. The producer should be free—as long as he practices the researched withdrawal periods on drugs, he can use as much as he wants. There should be no issues” (P8). Another farmer questioned the sensitivity of the meat and milk withdrawal times. “Our milk tests and meat tests, are they good enough in this country?” (P25). Even though the response to the interview itself served as support for the lack of clarity surrounding the connection between AMU in animals and concerns of AMR in humans, one farmer clearly articulated that, “I think it comes down to the science of it. Like, where is the problem exactly?” (P18).

### Theme 4: Dairy Farmers’ Perspectives on Antimicrobial Regulation

#### Subtheme 4a: Resistance to Increased Regulation as Fueled by Concerns of “Becoming Europe.”

When discussing AMU during a Saturday afternoon community gathering, one farmer stated, “We are becoming Europe.” Another shared, “The thing is, they keep saying, ‘This regulation program is going to be the answer.’ Now we have another regulation program coming in. Is it ever going to stop? No. We are going to get to be the Netherlands shortly” (P3). These concerns were consistently expressed in interviews and informal discussions. Many were specifically concerned that Canada might adopt protocols similar to those in some European countries, such as the requirement that a veterinarian administer all treatments, and that this would be expensive and reduce animal welfare. For example, “I hope we don’t come to the point of Europe and we need to have a vet to administer every drug, because I believe dairy farmers will lose a lot of money. If that ever happens, I don’t know if we will have enough veterinarians to do that. And then, I can see a lot of farms going the opposite way. I can see things getting really bad, because they won’t be treating sick cows” (P3). Farmers were similarly concerned about overall limits on AMU. One stated, “My biggest worry is if you do it the way Holland did it, where you limit how much you can use per year by limiting access, your animal welfare suffers” (P10).

Perceptions of farming regulations in Europe were not always explicit. One producer who stated that he would want to work to prevent Canada from taking a route where a veterinarian is needed for each treatment, also shared that he decided to switch from blanket to selective DCT based on European-based literature that outlined parameters to consider when deciding to treat. In his particular case, some aspects of European protocols were desirable, but, in other cases, too extreme. For example, he later stated, “I think Denmark is to the extreme” (P1).

Participants in the sample also expressed concerns regarding regulations that would require prescription per animal. One participant, whose parents farm in Holland, said, “It would be nice if we don’t get prescription per cow. Because that is how they do it in Holland now.” However, the same farmer also shared that we could learn from farmers’ experiences in Europe. In reference to switching to selective DCT, he stated, “In the beginning, there were big disasters [in Holland] and people said that it’s never going to work. And now, it seems like everybody’s doing it because they have to.” He added that, “If Holland and Europe do it now first, then we can learn from that. And if it works over there, we can also implement it here” (P9).

One family supported stricter regulations in Holland. A family member shared, “Sometimes the issue is that farmers don’t get pushed too hard to think differently…They’ve been pushing in Holland like crazy, and look, … they do better than us here. If you talk antibiotics, if you talk about environmental things… everything is recorded, from bringing manure to fertilizer on the ground, cows and milk you sell, it’s all on paper. So, the push for them to do better is harder.” Another member of this family added, “They’ve cut antibiotic use in half there… Maybe Europe is ahead a bit… trying to be proactive” (P5).

#### Subtheme 4b: Frustration with Impractical Policies.

Overall, farmers expressed great frustration with policies perceived as impractical or illogical, that do not create meaningful change at a societal level, do not improve on-farm management, or do not make economic sense. Many farmers spoke of the proAction Initiative (http://dairyfarmers.ca/proaction) and the Canadian Quality Milk Program (http://dairycheq.com/about/quality-milk/), 2 programs implemented by farmer-run organizations. Although most agreed these programs served a purpose, even if just to maintain consumer trust, some were displeased with specific requirements, especially paperwork, as it did not improve on-farm management. One farmer stated, “I can guarantee you one thing. If farmers created this program, there would be no paperwork. Or limited paperwork” (P3). It is noteworthy that by “farmers” he was referring to those farmers who actively participate in daily chores and on-farm work. Additionally, it is important that new regulations make economic sense and alternatives are provided. One farmer shared, “It has to make economic sense, and then after that you have to do what’s right. But, they have to come up with alternative[s] before they [implement] change[s]” (P9).

One farmer’s concerns were regarding the potential for arbitrary and absolute reductions in AMU and with the prospect of authorities not acknowledging previous efforts. He stated, “If we’re already treating case by case, how can you tell me, ‘Oh, you have to reduce it.’ Well, why do I have to reduce it from this point? What if I’ve reduced it already from 5 years ago? … It is hard when you’ve already reduced, it can be difficult to reduce more.” Regarding absolute reductions in use, he said, “… why should there be a set level? Why should we decide, ‘Okay, we are going to reduce 50%?’ How come 50% is okay? Why shouldn’t it be 75 or 25? It’s just a number somebody picks because it sounds good” (P4).

Whereas some of the farmers interviewed expressed concern about new AMU regulations, others did not appear to share these views and one stated, “I think too many people are always set in their ways. ‘This is how we’ve always done it.’…People have to start being more open to what’s out there…the farmer needs to be a little more proactive and say, ‘You know, the consumer’s demanding this and this.’ Maybe we just need to change and do [away with the], ‘This is the way we’ve always done it attitude.’ We need to change to a more progressive, open attitude” (P5).

#### Subtheme 4c: Antimicrobial Regulations Fueled by Consumer Perception and Not Science.

Some producers thought it was unlikely for increased regulation to be scientifically driven but rather to be influenced by misinformed consumers in urban centers with little knowledge of industry standards. One said, “[Consumers] influence policy and government…and that’s where all regulations come from…very little of it is science-based…I think everything we do should be completely science-based… And if we don’t do the scientific research, we don’t have anything to push with. But I don’t think it has near the influence or power that it should” (P4). Another acknowledged social pressure but did not think it affected regulations.

Another farmer was quite skeptical of underlying drivers to switch from blanket to selective DCT. He articulated that the primary impetus would be economic and to demonstrate reduced AMU to the public. However, he questioned the human health benefits, suggesting that, although potentially cost effective, it would not be a meaningful change and could be considered public deception. He cited recent work which suggested that intramammary use in dairy cows was not directly linked to AMR in humans. “I just think we can save ourselves some money doing it. It’s an economic reason for us. It’s not because of antibiotic usage. Because blanket dry cow treatment has nothing to do with resistance. Research has shown that blanket dry cow treatment is not related to resistance… There’s a public health perception, but it’s a false one” (P8). Regarding future antimicrobial regulation changes, he said, “If you want to affect numbers, go from blanket treatment to selective dry cow treatment. That will get huge effect on numbers. But will it have an effect on antimicrobial resistance?… I don’t think so. Not based on what the research says, anyways. It would be misleading. There would be a deception to the public if you went from blanket to selective to deceive the public into thinking antimicrobial usage is reduced in a way that will affect resistance. It will show a decrease, so the public might be happy, but it’s deceptive.” Regarding antimicrobial reduction, he later said, “I want to do it because it does something, not because it makes people feel good” (P8).

### Theme 5: Dairy Farmers’ Perspectives on Current AMU and Stewardship

#### Subtheme 5a: Antimicrobials as Tools with Value in Support of Animal Welfare.

Antimicrobials can be understood as valuable tools, to support animal welfare. Some dairy farmers shared concerns of the negative effects that changes to AMU and regulation would have on animal health and welfare. For some, antimicrobials enabled them to act in the best interest of the animal and as dairy farmers, they considered that their responsibility, as the animal cannot act in its own best interest. Many expressed frustrations with “antibiotic-free” campaigns, as they implied that all other animal products contain antimicrobials and abstaining from AMU is preferred. One participant shared that the message implicit in this advertising does not recognize that when a cow is sick, she deserves to be treated. Another shared, “…You can’t tell an organic guy who’s got a pneumonia cow or a cow with mastitis, ‘No, you can’t use any antibiotics.’ …I think that’s an animal welfare issue…As dairymen, we have to do what we can to save an animal…” (P1).

However, not all were concerned with what the prospect of increased regulation or pressure for reduced use would mean for animal welfare. Instead, one family attributed poor management and subsequent effects on animal welfare as related to increased AMU. They shared that the issue was not AMU. The issue was management and the demands of animal production. They stated, “I think if we keep our cows in the situations they are in today, on concrete, on rubber mats, in the barn all the time, and on silage all the time, I don’t think you can reduce [AMU] by much” (P5). If regulations increased, they thought it would encourage farmers to consider other options “outside the antimicrobial box.” In reference to increasing regulation, they noted, “…farmers [would] have to think harder how to solve a problem.” They also added, “When there’s a Band-Aid solution, people can let things go…But when you know there are those regulations, I think there will be more people who will [take] … more preventative [steps]” (P5).

#### Subtheme 5b: Antimicrobials Provide a Sense of Safety, Act as an Insurance Policy.

Antimicrobials are arguably also a tool to deal with uncertainty or unpredictability. For example, one farmer noted that disease outbreaks can be unexpected. “I think most farmers already would like to reduce as much as they can…; but you never know what happens, right? That’s the problem, you never know what’s coming next. You might try to line everything up well and then something unexpected happens. You get a whole bunch of pneumonia or you get diarrhea” (P7).

Blanket treatments (e.g., DCT or calf pneumonia) can serve as a safety net. Regarding blanket DCT, one farmer shared, “Animal welfare-wise, I think optimal is still blanket treatment because you have no holes in the wall…If you look at blanket treatment as a big wall to help prevent mastitis, you have no holes there, nothing gets through because it’s a complete wall, right?” (P8) When discussing blanket treatments, it was clear that for some farmers it provided a sense of safety, assuring them that their cows were protected against disease and they against financial losses. One farmer mentioned that although blanket treating acts as insurance, it may be unnecessary. He stated, “… On one side, it’s kind of a blanket insurance … and on the other hand, it just doesn’t make a whole lot of sense…You do a lot of things sometimes to make sure nothing happens, to protect the animal…not really knowing, if you move away from this, is it going to really be a problem? Is it not?…That’s why it kind of gives me a better feeling [to use it]” (P6).

Ceftiofur (Excenel, Zoetis) is a third-generation cephalosporin. Third-generation cephalosporins have been identified as high-priority critically important antimicrobials for human health, due to limited alternative therapies, particularly for the treatment of *Salmonella* spp. and *Escherichia coli* infections in children (World Health Organization, 2018). It is a significant antimicrobial in this context and was discussed frequently in interviews. Similar to blanket treatments, it provides a sense of safety due to its label for zero-day milk withdrawal, preventing expensive errors and discarded milk. Although most farmers considered this antimicrobial expensive, many felt that it was cost effective, due to avoidance of discarded milk. One farmer noted that his family members who farm in Holland were surprised with the quantity of Excenel used in Canada. He later stated that if required to stop use, he would oblige, but would need to be informed of a suitable alternative and his veterinarian would need to provide advice. The need for alternatives to ceftiofur was echoed by other farmers in the sample. In response to potential ways to improve antimicrobial stewardship, one farmer stated, “Maybe quit using Excenel…We would have to discuss alternatives…And that one, I could see us changing for public perception…If there aren’t [alternatives], then research needs to be done to find alternatives before banning the drug. Because if they ban the drug and there are no alternatives in place, then animal welfare is impaired” (P8).

#### Subtheme 5c: The Role of the Veterinarian in Antimicrobial Stewardship.

Although farmers in this sample generally value their autonomy regarding on-farm decision-making, it was evident that the herd veterinarian plays a significant role in advising the farmer related to AMU. The influence of the veterinarian was expressed surrounding decisions related to on-farm culturing and transitions to selective DCT. For example, one farmer states, “Based on the vet’s workshops and recommendations, we started doing selective [DCT]” (P18). Additionally, when discussing ways to improve stewardship, the farmers shared that the input of the veterinarian would be critical in creating awareness around AMU and in the development of sustainable changes to current AMU protocols. Feedback from the herd veterinarian would be welcomed by the farmer in scenarios where judicious use could be improved and would be preferred over alternative methods of enforcing more prudent use. A farmer explains, “I think if you have an active role with your vet, I think it is kind of on the vets and you to talk about it. [The vet can say], ‘Hey, you know you are buying a lot of antibiotic? What’s going on?’ …Yeah. I don’t mind moving to that route” (P15).

## DISCUSSION

The purpose of this study was to gain a contextualized understanding of Albertan dairy farmers’ perceptions of AMU and prospective AMU regulation. We sought to achieve this objective via ethnographic fieldwork that used semi-structured interviews in combination with direct and participant observation. Four of the most salient takeaways are: (1) Most farmers in this sample immigrated from or were the direct descendants of those who immigrated from the Netherlands or Switzerland. The underlying drivers of this migration and the lived experience surrounding it play a key role in their perceptions of future AMU regulation; (2) They highly value their autonomy; (3) We observed variable skepticism in the farming community about the link between AMU in dairy cattle and AMR in humans; and (4) We detected concern that prospective regulations will be driven by misguided consumers, rather than scientifically supported evidence. In this section, we consider those takeaways as informed by ethnographic fieldwork conducted by the first author (JAI) and in light of related work abroad and in Canada.

Upon entry into the community, it was immediately apparent to the first author (JAI) that the community maintained extensive ties to its European roots. The historical, cultural, and familial connections of the community to the Netherlands (and in 3 cases, Switzerland) was apparent throughout the fieldwork and informed the dairy farmers’ beliefs concerning AMU in Alberta. For some individuals in the sample, having a previous context by which to reference provided a comparison for changing AMU regulations in Alberta. For example, as stated in the results, the perspectives of those who had family members already practicing selective DCT in the Netherlands were influenced by the experiences of their family members with the adoption of these practices. In some ways, this positively affected their perceptions, allowing them to see the feasibility of the practice, while also giving them insight into the potential pitfalls that may arise during the adoption of new practices.

Insights gleaned from the fieldwork revealed that the desire for autonomy stemmed from their collective lived experience as dairy farmers in the Netherlands and Switzerland. Even though not all participants emigrated from Europe, the cultural diffusion of this value was pervasive in the community and arguably underpins the community’s concern about specific AMU regulations that would restrict their autonomy. In reflecting upon the fieldwork, the Albertan dairy farmer in this community felt encroached upon in [their] farming past. As described by those who emigrated from the Netherlands, there was not much land. One farm was positioned between 2 others, leaving little space for expansion. Regulations on farming were increasing and authorities had more of an influence over on-farm practices. Farming no longer represented a livelihood that was exciting. At that time, a dairy farming future in Alberta offered the potential for independence, the propensity for financial gain and stability, land, and the opportunity to achieve shared goals, yet do so in unique, creative ways that were farm- and family-specific.

Similar to dairy farmers in the Netherlands (Kramer et al., 2017), there is concern about future antimicrobial regulation and community members hope antimicrobials will remain available to them. The desire for autonomy will undoubtedly affect the uptake of new practices in Alberta and their perception of future regulation. As described in the results, regulations that threaten to restrict their autonomy, such as policies that (1) place restrictions on antimicrobials with zero-day milk withdrawal, while offering no suitable alternatives, or that (2) require prescription per animal or (3) a veterinarian to administer each dose, would not be well-suited for this community and would ultimately serve to hinder research and policy initiatives aimed at improving antimicrobial stewardship.

However, other aspects of the social fabric of this community (e.g., religion) create an already present foundation for antimicrobial stewardship. For community members who identified as stewards of the land or maintained strong ties to the religious community or a connection to a higher power, antimicrobial stewardship was a natural extension of their current value system. Their motivation for farming, responsibility for careful use of resources, and care for animals, humans, and the environment suggests that prudent use of antimicrobials is desired and meaningful to the community.

In reflecting upon the interview process and informal discussions had in the context of the ethnographic fieldwork, the interviewer (JAI) came to understand that the interview itself revealed an important gap. That is, the conviction of the responses regarding questions about milk safety protocols brought to light the misconception among farmers of the underlying pathways of acquiring AMR. More specifically, many farmers assumed that the global health challenge was exclusively due to contamination of the food supply (e.g., the milk supply), but seemed to be unaware of other pathways that may increase AMR (i.e., environmental contamination via run-off or manure on crops). Therefore, the incongruency, at least partially, lies in the misguided notion that AMR has become a burden for humans strictly due to eating unsafe food. The lack of clear information regarding the pathways has the potential to lead to substantial frustration, and worse, skepticism regarding the link between AMU in dairy cattle and AMR in humans, on the part of the farmers, who feel they are working diligently to secure the safety of the milk supply. Skepticism regarding the link between AMU in agriculture and AMR in humans has also been noted by other studies, in which only 10% of Canadian respondents and 6% of farmers in England and Wales agreed that AMR in humans is linked to AMU in agriculture (Young et al., 2010; Jones et al., 2015). The lack of clear information provided to dairy farmers can undermine best efforts to improve antimicrobial stewardship. Therefore, a key point for future intervention is further explanation, to the extent that it is known, of the mechanism(s) by which AMU in dairy farming poses a risk for the development of AMR in humans, recognizing that the scientific link is complex.

Further clarification of the above-mentioned point may also work to lessen farmers’ concerns of prospective AMU regulation being driven by uninformed consumers and not based on scientific evidence, an idea which likely stems from the perceived ongoing mutual distrust between the farmer and the public. The perceived distrust was also evident via informal discussions throughout the ethnographic fieldwork in which the consumer was described as an urban dweller, far both geographically and due to the existing emotional and knowledge gaps from the farmer. Similar to the emotional gap present between society and dairy farmers in Germany, the Netherlands, and Sweden (Swinkels et al., 2015; Fischer et al., 2019), those in our sample are concerned that the consumer has little knowledge or appreciation of daily on-farm occurrences, on-farm decision-making processes, and the extensive knowledge and expertise of the farmer. The line between self and “other” appeared to be stark, with local political movements to push against farming land restrictions serving as a manifestation of such distrust.

Although recent work conducted with dairy farmers in Ontario and New Brunswick suggests there are several views concerning AMU reduction among dairy farmers in that context, some ideas were expressed more consistently and there is notable overlap with the findings of the current study (Cobo-Angel et al., 2021). Farmers in Ontario and New Brunswick expressed concerns that there is the expectation of reducing use placed on them by both consumers and the general public. However, they are skeptical of whether these 2 stakeholders are informed about the daily work and responsibilities involved in dairy farming. This potential disconnect may be leading to the expectation of reduction. Other farmers shared that they were unlikely to implement changes related to AMU reduction without new policy that enforced it, an idea also expressed by several participants in this study (Cobo-Angel et al., 2021). On the contrary, one key aspect of the context, that is, the supply management system, may help to promote antimicrobial stewardship. As learned via the ethnographic fieldwork, the supply management system creates economic stability and allows for a more resilient industry. Therefore, farmers may be more willing to engage in new practices with uncertain outcomes, such as ones that include more prudent AMU.

The role of the veterinarian in on-farm decision-making has been made clear by previous studies in Alberta as they are one of the key sources via which farmers gain new information (Ritter et al., 2015). Relationships with herd veterinarians are therefore also an important aspect of the context. Based on ethnographic observations, the veterinarians of farmers in this sample previously did not play a strong role in encouraging farmers to more prudently use antimicrobials. However, insights gleaned through the fieldwork suggested that more recently they have begun to encourage on-farm bacterial culture before treatment and support the switch from blanket to selective DCT, which in the case of at least one veterinarian was surprising to his clients as he previously advocated for blanket DCT. Although farmers in this sample were largely autonomous actors, it was clear that the veterinarian was a trusted member of the operation and advice from the veterinarian would be thoughtfully considered and welcomed. Additionally, given the lack of trust in policies perceived to be consumer-driven, buy-in from herd veterinarians would likely be required to promote farmer adherence to antimicrobial recommendations.

This work seeks to understand the variables which influence farmers’ decisions about AMU. Useful here is work by behavioral economist and psychologists on decision-making, including, notably, that of Kahneman and Tversky (1979) and Thaler (1980, 1985). These authors point out that until relatively recently, many behavioral scientists subscribed to utility theory, a view that holds that people are rational actors who make decisions strictly in accordance with logic and reasoning. Work by Kahneman and Tversky originating in the 1960s, Thaler in the 1980s, suggested otherwise: people are only rarely rational actors, a finding known as prospect theory. Prospect theory has been subsequently supported by a robust body of work in the social sciences (Brosnan and De Waal, 2003; Sapolsky, 2017). In short, this work finds that utility theory is myopic in its failure to take into account the role of other factors that shape decisions, including emotion and broader cultural influences. Hence, prospect theory tells us that the assumption by veterinarians and public health experts that dairy farmers will follow their recommendations as it “makes logical sense” is likely misguided. Rather, these recommendations will be considered by farmers in light of their personal experience, emotions, and cultural context. Given this, the advice provided by public health experts and veterinarians may well be useful and the dairy farmer may well have a complete understanding of the rationale for the advice, but these 2 facts alone may have little effect on their behavior.

Farmers in this study express a range of views about dairy farming in general and AMU in particular. With knowledge of these views in hand, policy makers concerned about AMU may more effectively address their concerns. For example, these policy makers may endeavor to provide a clear summary of the state of the science on the relationship between AMU in dairy cows and AMR in the human population. Such a summary should note that the authors appreciate the dairy farmers’ skepticism. As well, policy makers may find dairy farmers more receptive to policy if they take the time to meet with the farmers to appreciate their knowledge and training, dedication to their work, and their approach to on-farm decision-making.

By elucidating the relevant perceptions, lived experiences, and values of the community, we are better positioned to develop AMU policies that are context-specific and therefore, sustainable, and effective within a given community. More broadly, this work serves to further the development of the integration of social science and veterinary medicine under the One Health paradigm. The potential implications of this integration are widespread; the integration, and more specifically the implementation of anthropological tools, can be used to further our understanding of the social contexts surrounding emerging veterinary medical health challenges across all food production sectors. By expanding our understanding of these contexts, we are working to secure the health of human, animal, and environmental communities affected by these systems.

### Strengths

Semi-structured interviews and ethnographic fieldwork (i.e., direct and participant observations) were used to uncover dairy farmers’ perceptions surrounding AMU, AMR, and regulations. These perceptions, engendered by sociocultural and political-economic contexts, are essential to understanding the behavior of AMU. The ethnographic fieldwork component is one of the greatest strengths of the study, as it allowed the researcher to build relationships and trust within the community, which is not feasible through most other methods. Many studies on this topic used surveys (Young et al., 2010; Jones et al., 2015; Vasquez et al., 2019). However, this study used semi-structured interviews, which enabled farmers to express themselves in their own way and to respond to topics of greatest interest. Semi-structured interviews also allowed for probing by the researcher, which would not be available in a survey. As AMU is considered a sensitive topic in agriculture, it is possible that participants may provide socially desirable responses, particularly via survey response, where there is no rapport between the researcher and participant. However, because the current interviews were conducted within the context of ethnographic fieldwork, it is likely that the farmers felt more comfortable to openly share their thoughts. Additionally, insights gleaned through the fieldwork were used to contextualize the perceptions shared via the interviews.

### Limitations

A strength of ethnographic research is the depth of understanding it can provide, but this also results in limitations. In light of this, we suggest quantitative analyses be conducted with increased sample size to test the hypotheses that arose from this study. As well, because snowball sampling was used and participants primarily consisted of dairy farmers in a small area, it cannot be assumed that the results of this study are generalizable to all dairy farmers in Alberta. It is also possible that the interviewed farmers differed in their perceptions of AMU and AMR from those that did not agree to participate, leading to selection bias. Finally, interviewer bias may have been introduced via the unintentional use of leading questions, and it is also possible that the researcher’s perceptions on the topic may have influenced identified themes.

## CONCLUSIONS

Many dairy farmers in this sample (1) value their autonomy and hope to maintain agency regarding AMU; (2) have shared cultural and immigrant identities which may inform their perspectives of future AMU regulation as it relates to their farming autonomy; (3) feel that certain AMU policies implemented in other contexts would be impractical in Alberta and would constrain their freedom to make what they perceive to be the best animal welfare decisions; (4) believe that their knowledge and experience are undervalued by consumers and policy makers; (5) are concerned that the public does not have a complex understanding of dairy farming and, consequently, worry that AMU policy will be based on misguided consumer concerns; and (6) are variably skeptical of a link between AMU in dairy cattle and AMR in humans due to their strict adherence to milk safety protocols that is driven by their genuine care for the integrity of the product. The current study begins to consider contextualization of AMU in the dairy industry. Future studies on this topic would benefit from a deeper understanding of societal and cultural infrastructures that support AMU and of the culture of intensive dairy farming. Society would likely benefit more from a greater emphasis on increasing antimicrobial stewardship than it would from absolute reductions in AMU. Finally, future research should be directed away from educational interventions as previous health research has already made evident that awareness does not necessarily lead to behavior change. Instead, research on how best to use context-specific information to inform policy on AMU might be an area for further development.

## Figures and Tables

**Table 1.** Descriptive summary of personal attributes and background of dairy farmers included in the study

Farm	No. interviewed participants	Age (yr)	Sex (M = male; F = female)	Post-secondary education (Y = yes; N = no)	Dairy farming background	Immigrated to Canada (Y = yes; N = no; P = parents; G = grandparents; GG = great-grandparents immigrated)
P1	1	39	M	Y	20 yr farming; can be traced back 2 generations; father and grandfather farmed	P (father immigrated from Netherlands)
P2	2 (husband, wife)	37	M	Y	1 participant: 16 yr farming; can be traced back 2 generations	Y (18 yr ago; Netherlands)
P3	4 (husband: primary interviewee; wife, father-in-law, mother-in-law also present and engaged)	31	M	Y	10 yr farming; can be traced back 3 generations	N
P4	1	37	M	Y	Farming entire life; can be traced back 1 generation	P (39 yr ago; Switzerland)
P5	3 (husband, wife, husband’s father, equal participation)	30; 29; 61	M; F; M	Y; Y; Y	Farming 10, 1, and 43 years, respectively; can be traced back 1 generation	2 participants: Y (17 yr ago; Netherlands); 1 participant: N
P6	1	49	M	Y	Farming entire life; can be traced back 4 generations	Y (26 yr ago; Switzerland)
P7	1 (wife was primary interviewee; husband present)	41	F	Y	23 yr farming; can be traced back before 1600	Y (23 yr ago; Netherlands)
P8	1	32	M	Y	10 yr farming; can be traced back 4 generations	Y (28 yr ago; Netherlands)
P9	1	44	M	Y	20 yr farming; can be traced back to 1950s; grandparents and parents; parents started in 1972, still farming in the Netherlands	Y (22 yr ago; Netherlands)
P10	1	29	M	Y	Can be traced multiple generations from both maternal and paternal side	Y (23 yr ago; Switzerland)
P11	1	37	M	Y	15 yr farming; from dairy farming background	Y (12 yr ago; Netherlands)
P12	1	38	M	Y	17–18 yr farming; can be traced back 3 generations	Y (21 yr ago; Netherlands)
P13	2 (husband, wife)	38; 38	M; F	Y; Y	18 yr farming; can be traced back 3 generations for 1 participant	1 participant: GG (Netherlands)
P14	1	41	M	Y	12 yr (own) and 20 yr total experience; can be traced back to start of family history	Y (1.5 yr ago; Netherlands)
P15	1	31	M	Y	7 yr farming; can be traced back 3 generations; immigrated from Holland	G (Netherlands)
P16	1	42	M	Y	Farming entire life; can be traced back to start of family history	Y (38 yr ago; Netherlands)
P17	1	43	M	Y	18 yr farming; 3 generations farming	Y (38 yr ago; Netherlands)
P18	1	27	M	Y	2.5 yr farming	G (Netherlands)
P19	1	31	M	N	12 yr farming; can be traced a few generations on maternal side	Y (24 yr ago; Netherlands)
P20	1	53	M	Y	Farming entire life; can be traced 100+ years	P (Netherlands)
P21	2 (husband, wife)	62; 63	M; F	Y; Y	35–36 yr farming; can be traced 4 generations and 3 generations, respectively	Y (38 yr ago; Netherlands)
P22	2 (husband, wife)	57; 52	M; F	Y; Y	33 yr farming; can be traced back 3 generations	Y (20 yr ago; Netherlands)
P23	1	39	M	Y	Interviewee: 15 yr farming; farming part of heritage; spouse: farming entire life; can be traced back 3 generations	Interviewee: Y (15 yr ago; USA); Spouse: G (Netherlands)
P24	2 (husband, wife)	34; 31	M; F	Y	Full time since college; can be traced back 3 generations and 2–3 generations, respectively	Y (23 yr ago; Netherlands)
P25	1	36	M	Y	19 yr farming; can be traced at least 4 generations	Y (19 yr ago; Netherlands)

**Table 2.** Descriptive summary of 25 dairy farms included in the study

Farm	No. milking cows	No. farm workers^1^	Milking system	Bulk tank SCC (1,000 cells/mL)	Method of dry cow therapy^3^	Vaccinates herd^4^
P1	60	NF = 0; F = 5	Parlor, herringbone	100	S	Y
P2	85	NF = 1; F = 2	Parlor, double 8 parallel (switching to AMS)	125	B (would consider switching to S)	Y
P3	103	NF = 1; F = 4	Automated milking system	375	B	N
P4	90–100	NF = 1; F = 3	Flat (walk-through)	80–150	B	Y
P5	116	NF = 0; F = 4	Automated milking system	140	S	Y
P6	95	NF = 0; F = 4	Automated milking system	110	B (discussing S with family)	Y
P7	480	NF = 7; F = 2+adult children	Parlor, double 20 parallel	100–130	B (trying to switch to S)	Y
P8	400	NF = 9; F = 5	Parlor, parallel	200	B (would consider switching to S)	Y
P9	180	NF = 3; F = 1	Parlor, double 10 herringbone	233	B	Y
P10	110	NF = 1; F = 2	Automated milking system	200	S	Y
P11	300	NF = 10; F = 3	Parlor, double 10 parallel (rotary beginning March 2019)	150	B (previously S, would reconsider S)	Y
P12	155	NF = 1.5; F = 2	Parlor, double 10 parallel	100	B	Y
P13	60	NF = 0; F = 3	Automated milking system	250	B (previously S)	Y
P14	220	NF = 1; F = 1	Rotary	~120	S	Y
P15	99	NF = 1; F = 3	Parlor, swing 12 parabone	~120	B (previously S, plans to return to S)	Y
P16	110	NF = 1; F = 3 adults + 4 children	Automated milking system	250	S	Y
P17	110	NF = 1; F = 1	Automated milking system	180	B	Y
P18	480	NF = 8; F = 0	Rotary	140	S	Y
P19	175	NF = 1; F = 4	Automated milking system	160	B	Y
P20	210	NF = 6; F = 1 adult + 4 children	Parlor, double 10 parallel	175–225	B	Y
P21	150–155	NF = 1; F = 3	Automated milking system	300–320	S	Y
P22	160	NF = 1; F = 3	Parlor, double 10 parabone	150	S	Y
P23	157	NF = 7; F = 10+	Parlor, double 14 parallel	250	B	Y
P24	230	NF = 3; F = 0	Rotary	132	B	Y
P25	100	NF = 0; F = 4	Automated milking system	150	B	Y

1 NF = nonfamily members; F = family members.

2 Self-reported bulk tank SCC (10^3^ cells/mL).

3 B = blanket dry cow therapy; S = selective dry cow therapy.

4 Y = yes; N = no.

## References

[R1] Adam CJM, Fortané N, Ducrot C, and Paul MC. 2020. Transition pathways toward the prudent use of antimicrobials: The case of free-range broiler farmers in France. Front. Vet. Sci 7:548483. 10.3389/fvets.2020.548483. 33134347 PMC7577212

[R2] Alberta Milk. 2022. Accessed Aug. 20, 2022. https://albertamilk.com/.

[R3] Bernard HR 2011. Research methods in anthropology: Qualitative and quantitative approaches. AltaMira Press.

[R4] Bernard HR, Wutich A, and Ryan GW. 2017. Analyzing qualitative data: Systematic approaches. 2nd ed. SAGE Publishing Inc.

[R5] Brosnan SF, and De Waal FBM. 2003. Monkeys reject unequal pay. Nature 425:297–299. 10.1038/nature01963. 13679918

[R6] Brown H, and Nading AM. 2019. Introduction: Human animal health in medical anthropology. Med. Anthropol. Q 33:5–23. 10.1111/maq.12488. 30811674 PMC6492111

[R7] Canadian Dairy Information Centre. 2020. Dairy barns by type. Accessed Aug. 20, 2022. https://agriculture.canada.ca/en/canadas-agriculture-sectors/animal-industry/canadian-dairy-information-centre/dairy-statistics-and-market-information/farm-statistics/dairy-barns-type.

[R8] Canadian Veterinary Medical Association (CVMA). 2021a. Establishing a valid veterinarian-client-patient-relationship. Accessed Aug. 20, 2022. https://www.canadianveterinarians.net/valid-vcpr.

[R9] Canadian Veterinary Medical Association (CVMA). 2021b. Veterinary oversight of antimicrobial use in animals in Canada. Accessed Aug. 20, 2022. https://www.canadianveterinarians.net/policy-advocacy/veterinary-oversight-of-antimicrobial-use-in-canada.

[R10] Census Profile. 2016. 2016 Census–Ponoka, Town [Census subdivision], Alberta and Alberta [Province]. Accessed Aug. 20, 2022. https://www12.statcan.gc.ca/census-recensement/2016/dp-pd/prof/details/page.cfm?Lang=E&Geo1=CSD&Code1=4808039&Geo2=PR&Code2=48&SearchText=Ponoka&SearchType=Begins&SearchPR=01&B1=All&GeoLevel=PR&GeoCode=4808039&TABID=1&type=0.

[R11] Chan KW, Bard AM, Adam KE, Rees GM, Morgans L, Cresswell L, Hinchliffe S, Barrett DC, Reyher KK, and Buller H. 2020. Diagnostics and the challenge of antimicrobial resistance: A survey of UK livestock veterinarians’ perceptions and practices. Vet. Rec 187:e125. 10.1136/vr.105822. 32948669

[R12] Charani E, Edwards R, Sevdalis N, Alexandrou B, Sibley E, Mullett D, Franklin BD, and Holmes A. 2011. Behavior change strategies to influence antimicrobial prescribing in acute care: A systematic review. Clin. Infect. Dis 53:651–662. 10.1093/cid/cir445. 21890770

[R13] Cobo-Angel C, Gohar B, and LeBlanc SJ. 2022. Values and risk perception shape Canadian dairy farmers’ attitudes toward prudent use of antimicrobials. Antibiotics (Basel) 11:550. 10.3390/antibiotics11050550. 35625194 PMC9137716

[R14] Cobo-Angel C, Leblanc S, Roche SM, and Ritter C. 2021. A focus group study of Canadian dairy farmers’ attitudes and social referents on antimicrobial use and antimicrobial resistance. Front. Vet. Sci 8:645221. 10.3389/fvets.2021.645221. 34212017 PMC8239135

[R15] Craddock S, and Hinchliffe S. 2015. One world, one health? Social science engagements with the one health agenda. Soc. Sci. Med 129:1–4. 10.1016/j.socscimed.2014.11.016. 25434985

[R16] Craig P, Di Ruggiero E, Frohlich KL, Mykhalovskiy E, White M, Campbell R, Cummins S, Edwards N, Hunt K, Kee F, Loppie C, Moore L, Ogilvie D, Petticrew M, Poland B, Ridde V, Shoveller J, Viehbeck S, and Wight D on behalf of the Canadian Institutes of Health Research (CIHR)–National Institute for Health Research (NIHR) Context Guidance Authors Group. 2018. Taking account of context in population health intervention research: Guidance for producers, users and funders of research. Southampton: NIHR Evaluation, Trials and Studies Coordinating Centre. Accessed Aug. 20, 2022. https://www.storre.stir.ac.uk/bitstream/1893/27205/1/Craig%202018%20context%20guidance%20FullReport-CIHR-NIHR-01.pdf

[R17] ESRC Working Group. 2014. Anti-microbial resistance: Setting the social science agenda. Accessed Aug. 20, 2022. https://www.primecentre.wales/resources/Anti-Microbial%20Resistance%20-%20Setting%20the%20Social%20Science%20Agenda.pdf.

[R18] Fischer K, Sjöström K, Stiernström A, and Emanuelson U. 2019. Dairy farmers’ perspectives on antibiotic use: A qualitative study. J. Dairy Sci 102:2724–2737. 10.3168/jds.2018-15015. 30612802

[R19] Friedman DB, Kanwat CP, Headrick ML, Patterson NJ, Neely JC, and Smith LU. 2007. Importance of prudent antibiotic use on dairy farms in South Carolina: A pilot project on farmers’ knowledge, attitudes, and practices. Zoonoses Public Health 54:366–375. 10.1111/j.1863-2378.2007.01077.x. 18035975

[R20] Gertz C 1973. The Interpretation of Cultures. Basic Books, Inc.

[R21] Gobo G 2008. Doing Ethnography. SAGE Publishing Inc.

[R22] Hammersley M, and Atkinson P. 2007. Ethnography: Principles in Practice. Routledge.

[R23] Higham LE, Deakin A, Tivey E, Porteus V, Ridgway S, and Rayner AC. 2018. A survey of dairy cow farmers in the United Kingdom: Knowledge, attitudes, and practices surrounding antimicrobial use and resistance. Vet. Rec 183:746. 10.1136/vr.104986. 30413678

[R24] Hockenhull J, Turner AE, Reyher KK, Barrett DC, Jones L, Hinchliffe S, and Buller HJ. 2017. Antimicrobial use in food-producing animals: A rapid evidence assessment of stakeholder practices and beliefs. Vet. Rec 181:510. 10.1136/vr.104304. 28847873

[R25] Jansen J, and Lam TJGM. 2012. The role of communication in improving udder health. Vet. Clin. North Am. Food Anim. Pract 28:363–379. 10.1016/j.cvfa.2012.03.003. 22664213

[R26] Jansen J, Renes RJ, and Lam TJGM. 2010. Evaluation of two communication strategies to improve udder health management. J. Dairy Sci 93:604–612. 10.3168/jds.2009-2531. 20105532

[R27] Jones PJ, Marier EA, Tranter RB, Wu G, Watson E, and Teale CJ. 2015. Factors affecting dairy farmers’ attitudes towards antimicrobial medicine usage in cattle in England and Wales. Prev. Vet. Med 121:30–40. 10.1016/j.prevetmed.2015.05.010. 26123631

[R28] Kahneman D, and Tversky A. 1979. Prospect theory: An analysis of decision under risk. Econometrica 47:263–291. 10.2307/1914185.

[R29] Kirchhelle C, Atkinson P, Broom A, Chuengsatiansup K, Ferreira JP, Fortané N, Frost I, Gradmann C, Hinchliffe S, Hoffman SJ, Lezaun J, Nayiga S, Outterson K, Podolsky SH, Raymond S, Roberts AP, Singer AC, So AD, Sringernyuang L, Tayler E, Rogers Van Katwyk S, and Chandler CIR. 2020. Setting the standard: Multidisciplinary hallmarks for structural, equitable, and tracked antibiotic policy. BMJ Glob. Health 5:e003091. 10.1136/bmjgh-2020-003091. PMC7513567 32967980

[R30] Kramer T, Jansen LE, Lipman LJA, Smit LAM, Heederik DJJ, and Dorado-García A. 2017. Farmers’ knowledge and expectations of antimicrobial use and resistance are strongly related to usage in Dutch livestock sectors. Prev. Vet. Med 147:142–148. 10.1016/j.prevetmed.2017.08.023. 29254712

[R31] Lam TJGM, Jansen J, van den Borne BHP, Renes RJ, and Hogeveen H. 2011. What veterinarians need to know about communication to optimise their role as advisors on udder health in dairy herds. N. Z. Vet. J 59:8–15. 10.1080/00480169.2011.547163. 21328152

[R32] Lam TJGM, Jansen J, and Wessels RJ. 2017. The RESET Mindset Model applied on decreasing antibiotic usage in dairy cattle in the Netherlands. Ir. Vet. J 70:5. 10.1186/s13620-017-0085-x. 28250915 PMC5322642

[R33] Laxminarayan R, Matsoso P, Pant S, Brower C, Røttingen J-A, Klugman K, and Davies S. 2016. Access to effective antimicrobials: A worldwide challenge. Lancet 387:168–175. 10.1016/S0140-6736(15)00474-2. 26603918

[R34] McCubbin KD, Anholt RM, de Jong E, Ida JA, Nóbrega DB, Kastelic JP, Conly JM, Götte M, McAllister TA, Orsel K, Lewis I, Jackson L, Plastow G, Wieden H-J, McCoy K, Leslie M, Robinson JL, Hardcastle L, Hollis A, Ashbolt NJ, Checkley S, Tyrrell GJ, Buret AG, Rennert-May E, Goddard E, Otto SJG, and Barkema HW. 2021. Knowledge gaps in the understanding of antimicrobial resistance in Canada. Front. Public Health 9:726484. 10.3389/fpubh.2021.726484. 34778169 PMC8582488

[R35] Opala J, and Boillot F. 1996. Leprosy among the Limba: Illness and healing in the context of world view. Soc. Sci. Med 42:3–19. 10.1016/0277-9536(95)00026-7. 8745104

[R36] Parkunan T, Ashutosh M, Sukumar B, Chera JS, Ramadas S, Chandrasekhar B, Ashok Kumar S, Sharma R, Santhosh Kumar M, and De S. 2019. Antibiotic resistance: A cross-sectional study on knowledge, attitude, and practices among veterinarians of Haryana state in India. Vet. World 12:258–265. 10.14202/vetworld.2019.258-265. 31040568 PMC6460864

[R37] Raymond MJ, Wohrle RD, and Call DR. 2006. Assessment and promotion of judicious antibiotic use on dairy farms in Washington State. J. Dairy Sci 89:3228–3240. 10.3168/jds.S0022-0302(06)72598-X. 16840641

[R38] Rees GM, Bard A, and Reyher KK. 2021a. Designing a national veterinary prescribing champion programme for Welsh veterinary practices: The Arwain Vet Cymru project. Antibiotics (Basel) 10:253. 10.3390/antibiotics10030253. 33802546 PMC7998244

[R39] Rees GM, Reyher KK, Barrett DC, and Buller H. 2021b. ‘It’s cheaper than a dead cow’: Understanding veterinary medicine use on dairy farms. J. Rural Stud 86:587–598. 10.1016/j.jrurstud.2021.07.020.

[R40] Ritter C, Kwong GPS, Wolf R, Pickel C, Slomp M, Flaig J, Mason S, Adams CL, Kelton DF, Jansen J, De Buck J, and Barkema HW. 2015. Factors associated with participation of Alberta dairy farmers in a voluntary, management-based Johne’s disease control program. J. Dairy Sci 98:7831–7845. 10.3168/jds.2015-9789. 26342983

[R41] Robinson TP, Bu DP, Carrique-Mas J, Fèvre EM, Gilbert M, Grace D, Hay SI, Jiwakanon J, Kakkar M, Kariuki S, Laxminarayan R, Lubroth J, Magnusson U, Thi Ngoc P, Van Boeckel TP, and Woolhouse MEJ. 2016. Antibiotic resistance is the quintessential One Health issue. Trans. R. Soc. Trop. Med. Hyg 110:377–380. 10.1093/trstmh/trw048. 27475987 PMC4975175

[R42] Sapolsky R 2017. Behave: The Biology of Humans at our Best and Worst. Penguin Press.

[R43] Schultz E, and Lavenda R. 2009. Cultural Anthropology: A Perspective on Human Condition. Oxford University Press.

[R44] Swinkels JM, Hilkens A, Zoche-Golob V, Krömker V, Buddiger M, Jansen J, and Lam TJGM. 2015. Social influences on the duration of antibiotic treatment of clinical mastitis in dairy cows. J. Dairy Sci 98:2369–2380. 10.3168/jds.2014-8488. 25682148

[R45] Tang KL, Caffrey NP, Nóbrega DB, Cork SC, Ronksley PE, Barkema HW, Polachek AJ, Ganshorn H, Sharma N, Kellner JD, and Ghali WA. 2017. Restricting the use of antibiotics in food-producing animals and its associations with antibiotic resistance in food-producing animals and human beings: A systematic review and meta-analysis. Lancet Planet. Health 1:e316–e327. 10.1016/S2542-5196(17)30141-9. 29387833 PMC5785333

[R46] Thaler R 1980. Toward a positive theory of consumer choice. J. Econ. Behav. Organ 1:39–60. 10.1016/0167-2681(80)90051-7.

[R47] Thaler R 1985. Mental accounting and consumer choice. Mark. Sci 4:199–214. 10.1287/mksc.4.3.199.

[R48] Tversky A, and Kahneman D. 1981. The framing of decisions and the psychology of choice. Science 211:453–458. 10.1126/science.7455683. 7455683

[R49] Vasquez AK, Foditsch C, Dulièpre S-AC, Siler JD, Just DR, Warnick LD, Nydam DV, and Sok J. 2019. Understanding the effect of producers’ attitudes, perceived norms, and perceived behavioral control on intentions to use antimicrobials prudently on New York dairy farms. PLoS One 14:e0222442. 10.1371/journal.pone.0222442. 31509595 PMC6738616

[R50] Vayda AP 1983. Progressive contextualization: Methods for research in human ecology. Hum. Ecol. Interdiscip. J 11:265–281. 10.1007/BF00891376.

[R51] Wall BA, Mateus A, Marshall L, Pfeiffer DU, Lubroth J, Ormel HJ, Otto P, and Patriarchi A. 2016. Drivers, dynamics and epidemiology of antimicrobial resistance in animal production. Accessed Aug. 20, 2022. http://www.fao.org/3/a-i6209e.pdf.

[R52] Woolhouse MEJ, and Ward MJ. 2013. Sources of antimicrobial resistance. Science 341:1460–1461. 10.1126/science.1243444. 24030495

[R53] World Health Organization. 2018. Critically important antimicrobials for human medicine: Ranking of medically important antimicrobials for risk management of antimicrobial resistance due to non-human use. Accessed Aug. 20, 2022. https://apps.who.int/iris/handle/10665/255027.

[R54] Young I, Hendrick S, Parker S, Rajić A, McClure JT, Sanchez J, and McEwen SA. 2010. Knowledge and attitudes towards food safety among Canadian dairy producers. Prev. Vet. Med 94:65–76. 10.1016/j.prevetmed.2009.11.010. 19962773

[R55] Zinsstag J, Schelling E, Bonfoh B, Fooks AR, Kasymbekov J, Waltner-Toews D, and Tanner M. 2009. Towards a “One Health” research and application tool box. Vet. Ital 45:121–133. 20391395

